# Coexisting of bone marrow fibrosis, dysplasia and an X chromosomal abnormality in chronic neutrophilic leukemia with CSF3R mutation: a case report and literature review

**DOI:** 10.1186/s12885-018-4236-6

**Published:** 2018-03-27

**Authors:** Xue Bin Wu, Wei Wei Wu, Yue Zhou, Xuan Wang, Jia Li, Yang Yu

**Affiliations:** Department of Hematology, Beijing Shijitan Hospital, Capital Medical University, Tieyi Road 10, Yangfangdian, Haidian District, Beijing, 100038 China

**Keywords:** Myeloproliferative neoplasm, Chronic neutrophilic leukemia, *CSF3R* mutation, Prognosis factors

## Abstract

**Background:**

Chronic neutrophilic leukemia (CNL) is a rare myeloproliferative neoplasm (MPN) with less than 40 cases of patients being reported or clinically suspected meeting with 2008 World Health Organization (“WHO”) diagnostic criteria. The current diagnosis of CNL remains to exclude other diseases. Recently, a new biomarker of *CSF3R* mutations that is almost invariably present in CNL has been identified. There is no effective treatment for CNL, therefore prognosis of the disease is poor, but it may be attributed to the presence of both *SETBP1* and *CSF3R* gene mutations. The presence or absence of *CSF3R* mutation did not affect survival, whereas a trend for shortened survival was observed among patients with *SETBP1*-mutation.

**Case presentation:**

Here we report a 65-year old woman patient who presented with leukocytosis without sign of fever and tumors. Bone marrow aspirates showed a markedly hypercellular feature with 76%–92% myeloid and the dysplastic changes were found in about 7% of neutrophils cells. The bone marrow biopsy demonstrated marrow fibrosis with Gomori staining positive (+++~++++). Cytogenetic analysis showed 46,X,del (X) (q22). No molecular markers of *BCR/ABL*1 rearrangement (P210, P230, P190 and variably), *JAK2V617F, FIP1L1-PDGFRA*, *TEL-PDGFRB, ZNF198-FGFR1* and *SETBP1* mutations were identified, however, the *CSF3R* gene membrane proximal mutation (c.1853C > T/p.T618I sites) was detected by PCR techniques. The patient was diagnosed with CNL and died in about 2 months after disease diagnosis.

**Conclusion:**

In clinical course, the CNL concurrently with severe bone marrow fibrosis and dysplastic features as well as X chromosomal abnormality may predict a worsening prognosis regardless of *SETBP1* mutation status.

## Background

Chronic neutrophilic leukemia (CNL) is an extremely uncommon myeloproliferative neoplasm (MPN) characterized by sustained, mature neutrophilic leukocytosis, hepatosplenomegaly, and bone marrow granulocytic hyperplasia. The key diagnostic criteria of CNL has been retained by the World Health Organization (“WHO”) for hematopoietic tumors [1]. However, a critical review of the literature using these WHO diagnostic criteria could only confirm 40 cases [2], representing approximately 51% of the clinically suspected patients with CNL [1], suggesting that the true rate of occurrence is even lower than suspected [2]. In 2013, a new biomarker of *CSF3R* mutations with a close association with diagnosis of CNL was identified [3–5]. In 2016,WHO revised the classification of MPN and added the *CSF3R* mutations as one of the CNL diagnostic criteria [6, 7].

The median age of CNL diagnosis is 66 years (range: 15–86), and the median survival of CNL is 23.5 months (range:1–106 months). The most frequent causes of death are intracranial hemorrhage, progressive disease or blastic transformation, and regimen-related toxicity from induction chemotherapy or transplantation [1, 2]. There is no effective treatment for CNL and therefore prognosis is poor. The poor prognosis factors of CNL are not clear. Recent research has showed that new biomarkers such as *SETBP1* and/or *ASXL1* mutations may be associated with poor prognosis in CNL and CMML [8], and presence of *SETBP1* mutation in CNL suggested a pathogenic role associated with progression of blast phase transformation [8]. Herein, we report a case with CNL who had a very poor prognosis due to concurrent of bone marrow fibrosis, dysplasia and an X chromosomal abnormality but without *SETBP1* mutation.

## Case presentation

Our patient is a 65-year-old Chinese woman with medical history of 1 year hypertension and 2 years intermission versatile skin purpura. Starting in February 2014, she had overt aggravated skin purpura with malaise, fatigues and anorexia. Peripheral blood count showed hemoglobin of 76 g/L, leukocytes of 72X10^9^/L with 92% neutrophils and platelet count of 41X10^9^/L. Peripheral blood smear examination showed no immature granulocytes and myeloblasts. The bone marrow aspiration revealed myeloid hyperplasia at 92%, and then she was diagnosed with CML-CP at a local hospital.

**Fig. 1:**
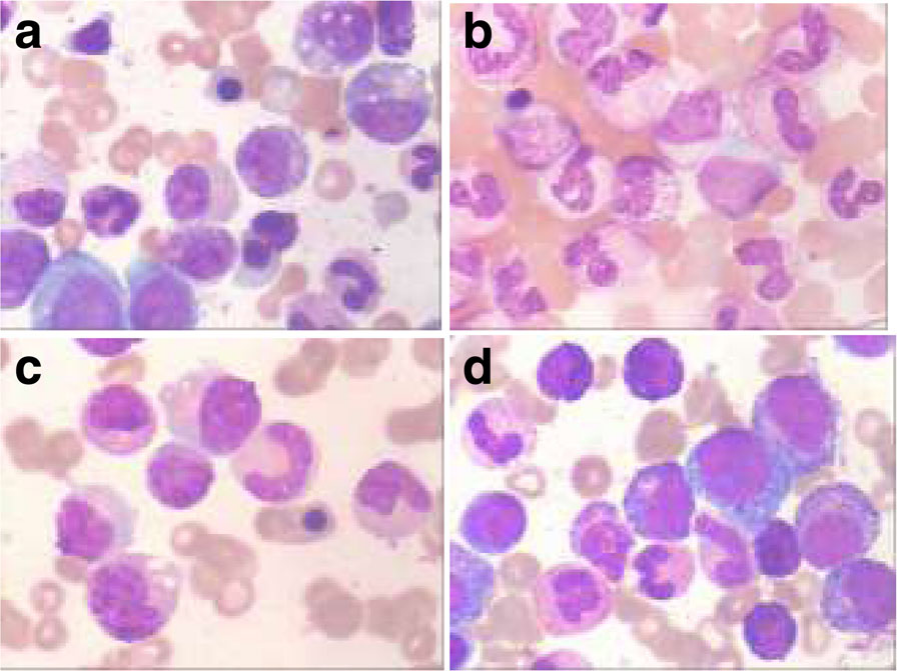
Bone marrow morphology. The morphology showed markedly hypercellular of granulopoiesis without increase in blasts. Dysplastic changes of neutrophils such as dikaryocyte, vacuoles, hypergranular cytoplasm, and hypersegmented nuclei were observed. **a** and **b**. Bone marrow and peripheral cells respectively in March,21; **c**. Bone marrow cells in March, 27; and **d**. Bone marrow cells in May, 14. (Wright–Giemsa stain, 1000×)

**Fig. 2:**
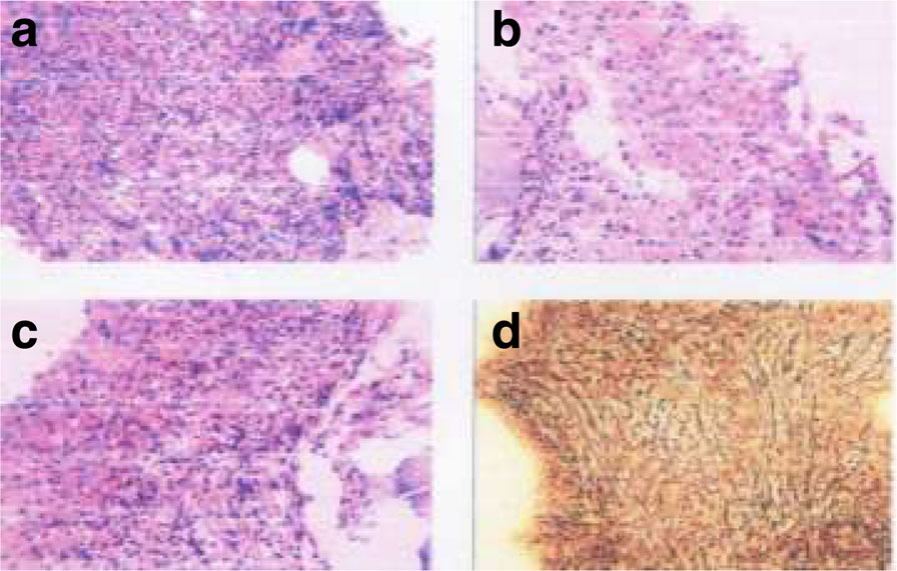
Bone marrow biopsy. Bone marrow biopsy examination showed markedly myeloid hyperplasia with focal fibroblasts and the Gomori staining was positive(+++~++++). **a**, **b** and **c** were HE stain(400×), **d** was Gomori stain(400×)

**Fig. 3:**
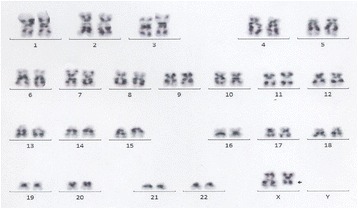
Karyotype of bone marrow cells. The karyotype showed 46,X,del(X)(q22), it was marked by an "arrow" in the picture. The conventional cytogenetic G-banding technique result of analysis was 46,X,del(X) (q22) [8]/46,XX[1]

She was admitted to our hospital on March 20, 2014. Physical examination showed no other positive signs except anemia. Complete blood count showed leukocytes of 85X10^9^/L with 90% mature neutrophils and 2% monocyte, there was no increase of blasts or immature granulocytes detected. The hemoglobin level was 74 g/L, platelet count was 62X10^9^/L. CT scanning showed mild splenomegaly, and B ultrasonography of the abdomen showed an splenomegaly (intercostal thickness was 5.5 cm).The lactate dehydrogenase (LDH) and creatinine was 499 U/L (40–240) and 70 umol/L (35–80) respectively. Bone marrow evaluation showed a markedly hypercellular with 76%–92% myeloid cells and the myeloid:erythroid ratio was 17.9, consisted of 1%–3% myeloblasts, 2%–8% promyelocyte, 1%–22% myelocyte, 6%–24% metamyelocyte, 30%–75% band and neutrophils, 2%–5% eosinophils, 0.4%–3% basophil, 1%–3% monocyte and 16% -18% erythroid cells. Dysplastic changes were found in about 7% of neutrophils such as dikaryocyte, vacuoles, hypergranular cytoplasm and hypersegmented nuclei (Fig. 1). The leukocyte alkaline phosphatase(LAP) score was increased to 387 (normal range 13–130) and the LAP positivity was 100% (Table 1). Immunophenotyping analysis of bone marrow cells by flow cytometry revealed that myelocytic cells consisted of 82%–90% with majority of CD16 + CD10+ mature granulocytes. The CD34 + CD117+ blast cells were 0.42%–0.6%, monocyte of 0.76%–4.06%, and few eosinophils, basophile and erythroblasts. Bone marrow biopsy examination showed marked myeloid hyperplasia with focal fibroblasts. Additional immunophenotyping of bone marrow cells demonstrated negative for CK, CD23, CD34, TdT and CD30, positive for VIM +, CD68 +, and EMA scattered +, CD3 scattered +, CD20 individual +, CD5 a few scattered +, MPO suffusion +, Ki-67(80%+), CD15 partial +, and the Gomori staining was positive (+++~++++) (Fig. 2). Conventional cytogenetic analysis showed 46,X,del (X) (q22) in 8 out of 9 metaphase spreads(46,X,del(X) (q22) [7, 8] /46,XX [1]) (Fig. 3). There were no molecular markers of *BCR/ABL*1 rearrangement (P210, P230, P190 and variably), *JAK2V617F, FIP1L1-PDGFRA*, *TEL-PDGFRB, ZNF198-FGFR1* and *SETBP1* mutations identified. However, the *CSF3R* gene membrane proximal mutation (c.1853C > T/p.T618I sites) was detected by PCR techniques. She was then diagnosed with chronic neutrophilic leukemia (CNL) and treated with hydroxyurea (1.0 g–2.0 g/day), EPO (6000 U/day) and Andriol (testosterone undecanoate, 160 mg/day). She was discharged on April 2nd of 2014, and her peripheral blood counts were leukocytes of 25X10^9^/L with 89% neutrophils, and Hb of 83 g/L and platelet of 25X10^9^/L.

**Table 1:** The results of the examinations and treatment courses of the patient

Date	Bone Marrow Cells			Peripheral cells			Treatment
	Myeloblast%	Dysplasia	LAP	WBC(X10^9^/L)	Hb(g/L)	Plt(X10^9^/L)	
21/03/14	1	granulocytes	387,100%	85,N:90%	74	62	Hu
27/03/14	2.6	granulocytes	NA	14, N:85%	60	29	Hu
31/03/14				25, N:89%	83	25	Hu
11/05/14				34,N:92%	62	3	Transfused
14/05/14	1.6	granulocytes	387,100%				Transfused
17/05/14				53,N:92%	65	5	Transfused
19/05/14				66,N:90%	60	21	Transfused
22/05/14				67,N:92%	56	11	Transfused
25/05/14				64,N:94%	67	5	Bleeding Transfused,
26/05/14				116,N:93%	34	7	Transfused
27/05/14				90,N:85%	33	29	Died

It displayed the examinations of bone marrow and peripheral cells at the process of the diagnosis and treatment courses of the patient

She was instructed to receive regular therapy of transfusion, hydroxyurea, EPO and Andriol. In late April, she experienced hematochezia. She was admitted again at our hospital on May 11, 2014. Physical examination showed an oral mucosa bleeding, blutpunktes and purpura on sporadic whole body, and both lower extremities oedema (+), without hepatosplenomegaly and superficial lymphadenopathy. Peripheral blood count showed leukocytes of 34X10^9^/L with 92% neutrophils and 3% monocyte, hemoglobin of 62 g/L and platelet of 3X10^9^/L. Bone marrow showed a markedly hypercellular with myeloid states cells and there was no increase of blasts (Fig. 1 and Table 1). PT: 12.6 s, PTA:78%, INR: 1.12, FIB:1.53 g/L, APTT: 26.5 s, TT:18.7 s, D-D:1026 ng/L, FDP:5.70 μg/ml, stool examination: bloody stool, occult blood(OB) (+), urine assayed: OB (+++). Her condition was slightly improved after standard treatment. On May 25, 2014, her disease worsened from lethargy to coma after a defecation. She had a hypomyodynamia and hypomyotonia in right limbs, and her right pupilla was larger than left one, concurrent with hematochezia, hematuria and hemoptysis. Peripheral blood count showed leukocytes of 67~116X10^9^/L with 85%~ 94% neutrophils but no immature granulocytes and myeloblasts. Laboratory examination showed the hemoglobin of 56 g/L and platelet of 11X10^9^/L. PT:12.4 s, PTA:75%, INR:1.12, FIB:3.56 g/L, APTT:25.2 s, TT:14.5 s, D-D:5243 ng/L. She was diagnosed with cerebral hemorrhage, gastrointestinal hemorrhage, pneumohemorrhage and respiratory failure. She passed away on May 27, 2014.

## Discussion and conclusion

CNL is a rare aggressive myeloproliferative neoplasm, similar to other MPN, the clinical course of CNL is heterogeneous. Clinical course of CNL is recognized by a chronic phase, an accelerated phase, and a blast phase respectively. Due to lack of specific molecular markers, the diagnosis of CNL has been considered of exclusion until an relevant CNL *CSF3R* mutation was identified in 2013 [1, 3]. Colony-stimulating factor 3 receptor gene (*CSF3R*) provides the proliferative and survival signal for granulocytes and also contributes to their differentiation and function. Nonsense somatic mutations affecting the cytoplasmic domain of *CSF3R* appear to be stem cell derived, associated with but not essential for severe congenital neutropenia -associated acute myeloid leukemia [4]. Maxson et al. identified activating mutations in the gene encoding the *CSF3R* in 16 of 27 patients (59%) with CNL or atypical CML [3]. Pardanani et al research showed that a total of 14 *CSF3R* mutations were identified in 13 patients, all of whom belonged to the group of CNL patients with either WHO-defined (*n* = 12) or unconfirmed (*n* = 1). The overall *CSF3R* mutational frequency was 100% in WHO-defined CNL and among these patients, the *CSF3RT618I* occurred exclusively in WHO-defined CNL with mutational frequency of 83% [4]. The current study was undertaken to determine the frequency, location and specificity of *CSF3R* mutations in CNL. A very strong association of mutations of *CSF3R* with WHO-defined CNL has been established and led to the reassessment of the current CNL diagnostic criteria. Presence of *CSF3RT618I* or other membrane-proximal *CSF3R* mutations have been recognized as one of the major diagnosis criteria at the revision of the WHO criteria for the diagnosis of CNL in 2016 [7]. According to this criteria, the CNL case presented here was diagnosed based on clinical manifestations as well as presence of *CSF3R* gene membrane proximal mutation [6, 7]. In the meantime, it has excluded other potential diseases such as infections, inflammatory conditions, solid tumors, and plasma cell neoplasm. The most frequent causes of death for CNL are intracranial hemorrhage, progressive disease/blastic transformation, and regimen-related toxicity from induction chemotherapy or transplantation [1, 2, 8]. Our patient had a fatal outcome due to a cerebral hemorrhage event. In addition, presence of *SETBP1* mutation may associate with a poor prognosis in CNL [8], however, our patient was absence of *SETBP1* mutation, therefore other pathological findings such as the bone marrow fibrosis and the dysplastic features of the bone marrow cells could be poor prognosis factors.

Presence of myelofibrosis is rare in CNL and it is important to distinguish between the primary and secondary fibrosis. Primary myelofibrosis (PMF) is a clonal hematologic malignancy with a variable disease course, its pathogenesis is associated with JAK2-STAT pathway and the somatic mutations included *JAK2V617F*, *MPL, CALR* and other epigenetic mutations (eg.*TET2, ASXL1* and *EZH2*) rather than *CSF3R* mutations [3, 4, 9–12]. Our patient had *CSF3R* mutation without *JAK2V617F*, thus it would support a diagnosis of CNL. Bone marrow fibrosis (BMF), which results from abnormal deposition of reticulin and collagen fibers in the bone marrow plays a major role in the pathophysiology and clinical manifestation of the disease and it has been suggested that BMF may affect overall survival in patients with myelofibrosis no matter primary and secondary fibrosis [12]. Our patient with BMF passed away died in less than 3 months after diagnosis. The presence of myelofibrosis in CNL suggests that the disease was evolving to the end-stage and it was reasonable to hypothesize that any disease evolution in CNL might be a worsening prognosis indicator [12]. Further clinical and biological studies and longer follow up are needed to fully understand the significant impact of BMF in patients with CNL.

Recently the *SETBP1* and *ASXL1* mutations were identified as prognostic biomarkers in CNL. Presence of *SETBP1* and *ASXL1* mutations have worse prognosis whereas *CSF3R* variants were not prognostically significant [13]. Pardanani et al. [4] research showed that *SETBP1* mutational frequencies in WHO defined CNL, aCML, CMML and PMF were 33, 0, 7 and 3%, respectively and concurrent of *CSF3R* and *SETBP1* mutations might have the worst prognosis for survival. However, the presence or absence of *CSF3R* mutation did not affect survival, whereas a trend for shortened survival was observed among patients with *SETBP1*-mutation alone [4]. Elliott et al detected *SETBP1*mutations in 5 out of 14 patients with *CSF3R*-mutated CNL and suggested pathogenetic roles for *SETBP1* mutation in disease evolution into blast phase disease of CNL [8], but another a meta-analysis shown that the *SETBP1* mutation was associated with a poor prognosis in patients with MDS and CMML, but not in patients with CNL [14]. Therefore, the further follow-up studies are necessary to confirm these findings so that *SETBP1* could be used as a prognostic marker to guide therapeutic decisions.

Cytogenetic abnormalities are found in about 23%~ 25% of CNL patients at diagnosis [2, 12]. These cytogenetic abnormalities are not to have significance for the diagnosis of CNL. Loss or gain of an X chromosome is usually accompanied by other chromosomal abnormalities and often associated with the hematologic malignancies such as acute leukemia, lymphoma and MDS, and also associated with dismal prognosis [15]. The X chromosomal abnormality is rarely found in CNL and only one case with an extra X chromosome was reported. This case progressed to blast crisis just in 2 months [16], so CNL with an X chromosomal abnormality may play an important role in the rapid progression.

Yamamoto et al. [16] reviewed 15 cases of CNL who terminated in the blast crisis and found 7 cases with dysplastic changes of granulocytes in the bone marrow cells. Among these cases, five had dysplasia in the erythroid and/or megakaryocytic lineage, as well as in granulocytic lineage. The median survival for 7 cases with dysplastic changes was 17.71 months, and for the other 8 cases without dysplastic changes was 54.62 months (*t* = 2.20586,0.05*>p>*0.02). Our patient with dysplasia in the granulocytic lineage survived in less than 3 months, thus, it is interesting to investigate if dysplasia might be an independently prognosis factor for the CNL. Note that utilization of novel biomarkers for CNL diagnosis were not widely accessible, therefore diagnosis of CNL remains to rely on morphology features of the bone marrow cells, such as bone marrow fibrosis and dysplasia in the CNL. The relationship between dysplasia and survival in CNL needs to be further explored in future clinical research. We hypothesize that coexistence of an X chromosomal abnormality, severe bone marrow fibrosis and dysplastic features of the bone marrow cells is one of the worsening prognosis factors no matter with or without *SETBP1* mutation. It is possibly the early signs of leukemic transformation for the CNL.

No standard of care exists for CNL. The primarily and the most commonly used treatment for CNL is hydroxyurea and/or interferon-α. These agents are effective in controlling leukocytosis and splenomegaly and maintaining a stable chronic phase, but not curable. No hematologic complete remission has been reported to date following standard induction therapy (anthracycline and cytarabine) for accelerated or blast phase in CNL [1, 7]. Given the potential for progressive refractory neutrophilia and blast transformation, allogeneic hematopoietic SCT has been performed in a number of cases and at this time represents the only known curative therapy [9]. Reviewing SCT in CNL patients, it is observed that 71% of the patients who received the transplant at the chronic phase have an ongoing remission of more than 7 months, in contrast with those who received it at the accelerated phase and died after the procedure [1]. Therefore SCT may result in favorable long-term outcomes in selected patients, particularly when undertaken in the chronic phase of disease [1, 7]. Optimal therapy of CNL remains to be defined, but significant advances are anticipated in the context of the recent progress in defining the molecular pathogenesis. *CSF3R* is known to signal downstream through both JAK and SRC tyrosine kinase pathways, and the two classes of *CSF3R* mutations exhibit different downstream signaling and kinase inhibitor sensitivities [1, 13]. Given the poor prognosis of this disorder, the potential applicability of JAK and/or SRC kinase inhibitors is another important implication of the discovery of activating *CSF3R* mutation. Treatment with the SRC kinase inhibitor Dasatinib and the JAK1/2 kinase inhibitor Ruxolitinib have shown efficacy for CNL with membrane proximal mutations and truncation mutations respectively in some patients [1–3, 7, 11]. These novel target-specific agents need further clinical investigation with larger CNL sample size.

CNL is still a deadly disease without effective diagnosis and treatment. *CSF3R* mutations have been confirmed as one of the WHO diagnosis criteria at the revision of CNL in 2016. Our patient had coexisting of the bone marrow fibrosis, dysplasia and an X chromosomal abnormality without *SETBP1* mutation led to a very poor outcome. This implies that CNL with severe bone marrow fibrosis and dysplastic features of the bone marrow cells as well as X chromosomal abnormality may predict a worsening prognosis of the clinical course regardless of the *SETBP1* mutation status. The recent developments in the knowledge of the molecular pathogenesis are the foundations for the identification of novel and effective therapeutic strategies.

## Abbreviations

aCML: atypical chronic myeloid leukemia
BMF: bone marrow fibrosis
CML-CP: Chronic myeloid leukemia -chronic phase
CMML: chronic myelomonocytic leukemia
CNL: Chronic neutrophilic leukemia
*CSF3R*: Colony-stimulating factor 3 receptor gene
EPO: Erythropoietin
MDS: myelodysplastic syndromes
MPN: Myeloproliferative neoplasm
PMF: Primary myelofibrosis
SCT: stem cells transplantation
WHO: World Health Organization
